# Clinical and biological impact of caffeine therapy in premature infants with apnea of prematurity: a prospective observational study

**DOI:** 10.3389/fmed.2026.1808401

**Published:** 2026-05-13

**Authors:** Adela-Valeria Neamțu, Ovidiu Mircea Zlatian, Andrei Theodor Bălășoiu, Costel-Valentin Manda, Carla-Maria Bărbulescu-Mateescu, Ana-Maria Pătrașcu, Ramona Cioboată, Liliana Stanca, Olivia Garofița Mateescu, Ștefan Pătrașcu, Antonia Radu, Simona-Daniela Neamțu

**Affiliations:** 1 Department of Anesthesia and Intensive Care, Doctoral School, University of Medicine and Pharmacy of Craiova, Craiova, Romania; 2 Department of Microbiology, Faculty of Medicine, University of Medicine and Pharmacy of Craiova, Craiova, Romania; 3 Medical Laboratory, Clinical Emergency County Hospital, Craiova, Romania; 4 Department of Ophtalmology, Faculty of Medicine, University of Medicine and Pharmacy of Craiova, Craiova, Romania; 5 Clinic of Ophthalmology, Clinical Emergency County Hospital, Craiova, Romania; 6 Department of Chemistry, University of Medicine and Pharmacy of Craiova, Craiova, Romania; 7 Faculty of Medicine, University of Medicine and Pharmacy of Craiova, Craiova, Romania; 8 Department of Hematology, Faculty of Medicine, University of Medicine and Pharmacy of Craiova, Craiova, Romania; 9 Clinic of Hematology, “Filantropia” Clinical Municipal Hospital, Craiova, Romania; 10 Department of Pneumology, Faculty of Medicine, University of Medicine and Pharmacy of Craiova, Craiova, Romania; 11 Clinic of Pneumology, “Victor Babes” Infectious Diseases Hospital, Craiova, Romania; 12 Department of Forensic Medicine, University of Medicine and Pharmacy of Craiova, Craiova, Romania; 13 Department of Histology, University of Medicine and Pharmacy of Craiova, Craiova, Romania; 14 Laboratory of Pathology, “Filantropia” Clinical Municipal Hospital, Craiova, Romania; 15 Department of Surgery, University of Medicine and Pharmacy of Craiova, Craiova, Romania; 16 Clinic of Surgery, Clinical Emergency County Hospital, Craiova, Romania; 17 Department of Pharmaceutical Botany, University of Medicine and Pharmacy of Craiova, Craiova, Romania; 18 Department of Immunology and Hematology, University of Medicine and Pharmacy of Craiova, Craiova, Romania; 19 Medical Laboratory, “Filantropia” Clinical Municipal Hospital, Craiova, Romania

**Keywords:** apnea of prematurity, hypoxia, LC-MS, methylxanthines, therapeutic drug monitoring

## Abstract

**Background:**

Methylxanthines are the first-line medication in apnea of prematurity. However, serum concentrations exhibit marked variability due to immature hepatic metabolism and drug–drug interactions.

**Objectives:**

This study aimed to evaluate premature newborns diagnosed with apnea of prematurity and highlight the role of therapeutic drug monitoring in preventing complications related to overaccumulation.

**Methods:**

This prospective observational clinical study was conducted on 325 premature neonates, divided into high caffeine citrate study group with serum titer >50 μg/mL (HC, *n* = 25) and normal caffeine group with serum titer <50 μg/mL (NC, *n* = 50) and a comparison group that did not receive caffeine citrate (CoG, *n* = 250). CoG required pulmonary ventilation, while HC and NC received both pulmonary ventilation and standard caffeine citrate. Serum caffeine citrate concentrations were monitored dynamically.

**Results:**

Major complications in caffeine group were intracranial hemorrhage (5.33%) and convulsions followed by death (4.00%) as, while the CoG demonstrated fewer adverse outcomes and more favorable clinical evolution.

**Conclusion:**

Intermittent administration of standard methylxanthine doses or adjustment of therapeutic doses, guided by serial monitoring of serum caffeine citrate titer, may improve clinical outcomes in critical cases, reduce adverse drug effects, and support safer and individualized therapeutic management of apnea of prematurity.

## 1 Introduction

Apnea of prematurity (AOP), cessation of breathing often associated with hypoxemia, cyanosis, and bradycardia, is a diagnosis of exclusion. An apnea episode is defined as an interruption of airflow for at least 20 s, frequently accompanied by a decrease in oxygen saturation and the appearance of cyanosis and/or a reduction in heart rate below 100 beats per minute (1, 2). In very small-for-gestational-age patients, desaturation and bradycardia can occur before 20 s of apnea and may raise the risk of neurodevelopmental impairment. Advanced pathological apnea is associated with pallor and hypotonia, and the newborn may not respond to tactile stimuli (3, 4). In preterm infants, apnea can affect mechanisms that protect cerebral blood flow, leading to ischemia and subsequent leukomalacia. Apnea episodes can also induce intestinal ischemia and possibly necrotizing enterocolitis by shunting cardiac output away from the mesenteric arteries in an attempt to preserve cerebral perfusion (5, 6).

From a pathogenic perspective, central apnea involves no inspiratory effort, obstructive apnea features ongoing inspiratory efforts with airway blockage, and mixed apnea the most frequent cause of apnea in preterm infants—occurs when airway obstruction with persistent inspiratory efforts either follows or precedes central apnea (7).

In around half of cases, apnea of prematurity results from both central and obstructive factors. This happens because premature infants have underdeveloped respiratory control in the brainstem, a weak response to vagal stimulation, and their pharynx can collapse due to negative pressure during inhalation. Additionally, the muscles that keep the airway open, such as the genioglossus and geniohyoid, are immature, making it harder for these infants to maintain airway patency. Once airway collapse occurs, adhesive forces at the mucosal level tend to prevent reopening during expiration (8, 9).

From an etiological perspective, apnea of prematurity is frequently secondary to maternal-fetal infections, hematological disorders in the newborn (including maternal-fetal incompatibility in the Rh/ABO system, corpuscular hemolytic anemias), prenatal exposure with transplacental transfer of various medications such as narcotics or beta-blockers, cervical incompetence, maternal metabolic diseases (such as diabetes mellitus or obesity), maternal endocrinological conditions (hypo/hyperthyroidism, autoimmune thyroiditis), thrombophilia, as well as maternal neurological, cardiovascular, and pulmonary diseases (10–12).

The delay in initiating breathing due to immature neural centers is more common with increasing degrees of prematurity. Brainstem function correlates with the onset of apnea episodes. As gestational age increases, the conduction time of the auditory evoked response shortens, and the frequency of apnea episodes decreases. The main mechanism by which methylxanthines reduce apnea consists of blocking adenosine receptors, thus reducing the hypoxic depression of respiration and increasing sensitivity to CO_2_ (13, 14). Sleep state influences the breathing pattern of the newborn. In preterm patients, rapid eye movement (REM) sleep predominates, associated with variations in respiratory rate and tidal volume. Apnea episodes are more frequent during REM sleep compared to quiet sleep (15).

Inflammatory biomarkers such as interleukins, tumor necrosis factor *α* (TNF-α), and procalcitonin (PCT) help evaluate and monitor preterm infants with apnea. Interleukin 6 (IL-6) regulates immune responses, PCT rises quickly in bacterial infections, and TNF-α indicates systemic inflammation. Combined with blood counts, these markers guide diagnosis and management of neonatal complications.

Understanding the underlying etiological and pathophysiological mechanisms of apnea of prematurity remains equally essential for clinical progress. Elucidating the roles of maternal-fetal factors, genetic predispositions, and the complexity of respiratory regulation at the brainstem level underscores the need for a holistic perspective on neonatal health. Furthermore, the precise characterization of inflammatory markers, including interleukins, procalcitonin, and TNF enhances early detection of complications such as sepsis and systemic inflammatory responses. Dynamic laboratory assessments, such as complete blood counts and biomarker profiling, complement clinical observations and support timely, evidence-based adjustments of therapeutic strategies.

Methylxanthines constitute a class of purine alkaloids characterized by their ability to antagonize adenosine receptors and inhibit phosphodiesterase enzymes. Blocking adenosine receptors facilitates the prevention of adenosine-mediated respiratory depression and increases the excitability of respiratory neurons. Phosphodiesterase inhibition contributes to increased excitability and improved respiratory drive by increasing intracellular levels of cyclic adenosine monophosphate (cAMP) which enhances the activity of protein kinase A, which phosphorylates target proteins involved in cellular excitation and neurotransmitter release (7). The evidence base for methylxanthine therapy in apnea of prematurity has progressed significantly over the past four decades (8). The landmark of Caffeine for Apnea of Prematurity (CAP) trial, published in 2006, confirmed the efficacy of caffeine in the treatment of apnea and demonstrated other benefits such as reduced bronchopulmonary dysplasia and improved neurodevelopmental outcomes. The CAP study established the reference regimen for caffeine citrate: a loading dose of 20 mg/kg (equivalent to 10 mg/kg caffeine base), followed by a maintenance dose of 5 mg/kg once daily, administered orally or intravenously. Optimal therapeutic plasma titers are 10–40 μg/mL, while levels above 50 μg/mL may cause toxicity. The therapeutic window for caffeine in premature newborns is relatively wide, but serum levels should not exceed 50 μg/mL, because above this threshold, adverse reactions may occur, such as tachycardia, agitation, convulsions, hydroelectrolytic and metabolic disorders (16, 17). The pilot trial by McPherson et al. in 2015 (17) testing a loading dose of 80 mg/kg, which demonstrated a higher incidence of cerebellar hemorrhage and abnormal neurological signs at term-equivalent age, raising safety concerns for supratherapeutic loading strategies. Nevertheless, a study by Moschino et al. (18) found no significant difference in neurodevelopmental outcomes at 2 or 5 years, indicating that higher doses do not confer neurodevelopmental benefit and may carry additional risk.

Medical information from clinical trials and pharmacokinetic data are limited, and personalized therapeutic approaches for premature infants who develop apnea of prematurity are lacking in the literature. Due to the paucity of research on caffeine citrate metabolism and the effects of drug combinations, especially their impact on hydro-electrolyte and metabolic imbalances with consequences on premature newborns, this study aims to fill this gap by correlating biological and clinical parameters throughout the patients’ evolution.

There are studies documenting marked variability in caffeine metabolism among individual preterm neonates (19), and the growing evidence base supporting individualized TDM-guided dosing, particularly in infants with sepsis, hepatic immaturity, or extreme prematurity. Emerging evidence suggests that intrauterine conditions, including placental insufficiency, may influence neonatal drug metabolism by modulating hepatic maturation. The research laboratories’ equipment with modern measuring devices allowed for the standardization of new techniques specific to the medical field. The bioanalysis of these compounds is becoming increasingly important. With advances in mass spectrometry, analytical laboratories have adopted highly sensitive detectors, which are more precise in assigning chromatographic peaks and more sensitive in analyzing low plasma concentrations (typically at the μg/mL level). Consequently, information regarding therapeutic drug monitoring (TDM) has become more accessible.

The study team conducted a comprehensive analysis of clinicopathological data regarding the possibility of improving the medical management system. The objective of our study was to document the interindividual variability of caffeine citrate by detecting serum titer in premature infants who developed apnea of prematurity and received routine treatment and to evaluate clinicobiological data to support the adjustment of therapeutic doses in order not to exceed the critical threshold of 50 μg/mL caffeine citrate. Avoiding the over accumulation of these drugs can be done by monitoring the serum titer over time, without exceeding the threshold of 50 μg/mL, either by intermittent administration of the usual doses, or based on personalized therapeutic doses (20).

The study also aimed to explore electrolyte and metabolic imbalances, inflammatory markers, hematological and placental histopathological changes and clinical outcome of infants in AOP.

## 2 Materials and methods

### 2.1 Study design and setting

#### 2.1.1 Participants

This was a prospective, single-center, observational clinical study conducted in a neonatal intensive care unit (NICU) on data collected from preterm infants. The aim was to describe the clinicobiological characteristics and short-term outcomes of preterm infants with clinically diagnosed apnea of prematurity and to assess how methylxanthine exposure recorded during routine care correlated with neonatal complications documented during the index hospitalization. This prospective observational clinical study with a comparison group was conducted between March 2024 and September 2025 at the Neonatology Department of the Filantropia Municipal Clinical Hospital, Craiova. The study involved 325 premature newborns aged 0–61 days without major congenital anomalies. Each underwent thorough clinical, paraclinical, and functional evaluations, performed by a multidisciplinary team, following established guidelines for prematurity. All participants met the study criteria for further analysis. We included in the study all pre-term infants which required respiratory support in NICU. Among the 325 pre-term infants included, 75 developed apnea of prematurity crisis, from which in 25 was detected at 24 h after the last therapeutic dose of high level of serum caffeine above 50 μg/mL (*HC group*), whilst the other 50 had serum caffeine levels below 50 μg/mL (*NC group*). 250 infants did not develop apnea of prematurity crisis and served as *comparison group (CoG group)*.

Cohort membership (exposure grouping used descriptively) was recorded in the electronic health record (EHR) as a variable labeled “Patient Group” (study vs. control vs. comparative). Demographic and perinatal characteristics included gestational age (weeks), sex, multiple gestation (twin pregnancy), 1/5 min Apgar scores, place of residence, and birth weight (g). All consecutive neonates admitted to the NICU during the study period were screened through the electronic health record. Data were stored on secure servers with access restricted to the study team.

##### 2.1.1.1 Ethical considerations

Between 01.10.2023 and 28.02.2024, the documents necessary for the issuance of the research project approval report by the ethics committee were submitted, and the inclusion of patients in the prospective clinical study began on 01.03.2024. The study complied with the Declaration of Helsinki and local regulations. The protocol was reviewed and approved by the institutional ethics committee of the Municipal “Filantropia” Hospital of Craiova (4,794/28.02.2024) and of the University of Medicine and Pharmacy of Craiova (no. 101/01.03.2024).

##### 2.1.1.2 Inclusion criteria

Participants qualified if: (a) the mother or guardian understood the national language and gave written consent; (b) prematurity was diagnosed with standard criteria; (c) a specialist neonatologist confirmed no major anomalies.

##### 2.1.1.3 Exclusion criteria

Participants were excluded if they had: (a) associated anomalies like Trisomy-21 or twin-to-twin transfusion syndrome; (b) respiratory or neurological conditions that could exacerbate apnea episodes; (c) mothers unable to understand or follow medical recommendations.

#### 2.1.2 General hypoxemia treatment

Premature infants included in the study developed cyanosis, hypoxemia, respiratory acidosis, and hypoglycemia, which led to admission to intensive care. Hypoxemia was managed with Neopuff ventilation, noninvasively Continuous Positive Airway Pressure (nCPAP Bubble) ventilation, or orotracheal tube intubation, under sedation with Midazolam during mechanical ventilation, with a maximum FiO2 of 80% and a gradual decrease in ventilation parameters, associated with the administration of a dose of intravenous Dexamethasone in order to wean from the mechanical ventilator.

Patients with respiratory distress syndrome (RDS) who required intubation received Curosurf (200 mg/kg), while hypoglycemia was treated with a glucose bolus and nutritional infusion. The neonates were then placed in closed incubators for thermal stability and oxygen therapy, with FiO2 reduced from 35 to 25%, along with partial parenteral nutrition.

#### 2.1.3 Methylxantines therapy (exposure)

Methylxanthine exposure followed the standard neonatal intensive care unit protocol: caffeine citrate was administered as a loading dose of 20 mg/kg (equivalent to 10 mg/kg caffeine base), then 5 mg/kg intravenously daily for maintenance. Apnea of prematurity episodes were treated with caffeine citrate for several consecutive days or intermittently until 37 weeks postmenstrual age. All patients received respiratory analeptics for a period of 5 to 18 days. Combination antibiotic therapy and concomitant methylxanthine administration were summarized, where available.

#### 2.1.4 Co-interventions and respiratory support

Delivery-room and NICU respiratory supports were abstracted from structured fields: pulmonary ventilation (VPP) Neo-Puff use and number of inflations, use and duration of nasal CPAP, need for oro-tracheal intubation and duration, and days of oxygen titrated in the blender (FiO₂). Concomitant use of antibiotics (gentamicin, meropenem, ampicillin, imipenem/cilastatin, vancomycin, amikacin, colistin, metronidazole, linezolid, ciprofloxacin, cefotaxime) and the total number used were documented to reflect illness severity.

#### 2.1.5 Outcomes

Outcomes were predefined, binary, and extracted from the electronic health record (HER) and daily notes using standardized variable labels. System-specific complications included: cardiovascular complications; perinatal asphyxia; respiratory complications; intraventricular hemorrhage (IVH); renal (acute) complications; fluid/electrolyte imbalance (hydroelectrolytic imbalance), defined per institutional laboratory reference ranges for sodium, potassium, chloride, and ionized calcium; ecchymosis and edema; allergic reactions and hematologic complications; vomiting, jaundice, heart murmur, convulsions; minor congenital findings (ankyloglossia, hydrocele, toe malposition, partial syndactyly, syndactyly agenesis, congenital dermal melanocytosis, short frenulum, hemangioma).

The short-term clinical progress was described using labeled categories: favourable, slow favourable, severe, and death, each represented as a binary indicator recorded throughout the hospital stay.

### 2.2 Methods

The newborns were examined and monitored using a clinical observation sheet, while laboratory investigations and imaging studies were performed at the Filantropia Craiova Municipal Clinical Hospital and the UMF Craiova Research Center. The novel methods for detecting drug concentrations in human serum, developed within the Research Center of the Faculty of Pharmacy in Craiova, were validated prior to their use to ensure adequacy and high accuracy of the results. The scope and precision of the values obtained through these validated methods are relevant to the needs of the beneficiaries (19). Validation was accomplished by assessing information gathered from existing literature and documentation provided by manufacturers regarding measurement equipment and reagents, by using reference materials in the laboratory, evaluating the uncertainty of results, and estimating factors that may influence outcomes.

Monitoring of serum caffeine citrate concentrations using validated LC–MS methods documented interindividual variability in drug metabolism.

#### 2.2.1 Caffeine measurements

Patient data include both serial measurements of methylxanthines and a binary indicator recorded as high caffeine level (>50 μg/mL).

Measurement of plasma caffeine levels was performed using an in-house, previously published LC–MS method in University of Medicine and Pharmacy of Craiova, Romania (19). Briefly, a simplified solid-phase extraction (SPE) approach was employed to prepare plasma samples (100 μL), which were subsequently analyzed using high-performance liquid chromatography coupled with mass spectrometry (LC–MS). Chromatographic separation was conducted on a CORTECS C18 column utilizing a water/acetonitrile gradient, with detection carried out in positive electrospray ionization mode. The developed method demonstrated sensitive and precise quantification capabilities, achieving a lower limit of quantification (LLOQ) at the μg/mL level. Comprehensive validation procedures confirmed the method’s accuracy, precision, and analyte stability (19).

Serum caffeine citrate levels were measured in 75 preterm infants 24 h after the loading dose, 24 h after the last therapeutic dose, 14 and 30 days after the end of treatment. Data included serial methylxanthine measurements and a binary marker for high caffeine concentrations (>50 μg/mL).

#### 2.2.2 Blood gas, and laboratory measurements

Pathophysiological data and laboratory results, including oxygen saturation, hemoglobin derivatives, arterial pH, pCO₂, pO₂, and bicarbonate concentration, were recorded in EHR flow sheets at routine NICU intervals. Laboratory tests included glucose, lactic acid, protein, bilirubin, complete blood count, blood smear cytology, and key electrolytes (Na^+^, K^+^, Cl^−^, Ca^2+^), reported both as continuous values and categorized by reference intervals. In the evaluation of preterm infants, we performed hematological, inflammatory, biochemical, and microbiological tests to assess infection risks and monitored oxygen saturation, arterial blood gases, and body temperature. Chest radiographs were recommended when tissue oxygenation was inadequate. Metabolic and intracranial pathology evaluations included serum methylxanthine, electrolytes, and glucose measurements, neurological examination, and transfontanellar ultrasound. (20–23).

Venous blood samples for hematology were collected in vacutainers containing either potassium or sodium EDTA powder. Blood cell counts and related tests were carried out with the automated Mindray BC 6800 Plus analyzer. To examine blood cell morphology, were studied using a Nikon Eclipse Ei microscope, first with a 40x dry lens and then with a 100x oil immersion lens, allowing for detailed analysis of different blood cells.

For testing inflammatory markers (such as C-reactive protein [CRP], IL-6, PCT, and TNF-*α*) and other biochemical measurements, blood was drawn into red-capped vacutainers without additives or yellow-capped ones with a gel separator and clot activator. After the sample clotted and was centrifuged, the serum was frozen at −20 °C. The levels of these serum substances were determined using the Maglumi X3 automated immunology analyzer, which uses chemiluminescence immunoassay (CLIA), and the Mindray BS-1000 M biochemistry analyzer. Biochemical testing methods included kinetic assays tracking changes in optical density, bi-chromatic endpoint photometry, and bi-chromatic two-point photometric techniques.

#### 2.2.3 Histopathological evaluation

Our study also included the microscopic evaluation of placental fragments corresponding to a gestational age of 25–37 weeks, collected at the Obstetrics and Gynecology Clinic of the Filantropia Municipal Clinical Hospital in Craiova. Within the Pathology Department of the same hospital, the collected fragments were processed by paraffin embedding technique and stained with hematoxylin–eosin. The placental fragments were initially fixed in 10% formalin. Subsequently, the technical steps for paraffin embedding were followed: dehydration, clearing, impregnation of the tissue with paraffin, actual embedding, sectioning, adherence of the sections to slides, staining with hematoxylin–eosin, which results in the cytoplasm staining pink and the nuclei blue-violet (23). The examination of the preparations was performed using a Nikon Eclipse-2 microscope equipped with an MSHOT-MSX2 camera, with image acquisition carried out using the MSHOT program. Detailed histopathological findings are provided in the Supplementary material.

#### 2.2.4 Statistical analysis

Analyses were performed using Stata software (version 17, StataCorp, College Station, TX, USA). Continuous variables are presented as mean ± standard deviation or as median and interquartile range, depending on distribution, with group comparisons conducted via Student’s *t*-test. Categorical variables are shown as counts and percentages and compared using the chi-square test or Fisher’s exact test when expected cell sizes were small. The main focus of the statistical analysis was the correlation of serum caffeine concentration with clinical and biological data of premature patients.

Clinical complications were coded as 0 or 1. Caffeine exposure was categorized as 0 for normal caffeine and 1 for high caffeine. For variables with multiple measurements (such as gases or electrolytes), data were harmonized based on NICU conventions—using either the first measurement after admission or the value deemed most clinically relevant. Data came from the institutional electronic health record and laboratory systems and were organized into a structured dataset. Prior to analysis, data were checked for completeness, valid ranges, and consistency.

To examine the relationship between levels of caffeine/xanthine exposure and clinical outcomes, we calculated odds ratios (OR) and 95% confidence intervals (CI) for various neonatal complications, comparing infants with normal versus high caffeine levels (>50 μg/mL), or comparing preterm infants without versus with apnea of prematurity.

Logistic regression models were used to estimate adjusted ORs with 95% CIs, treating high caffeine exposure as the primary predictor. Adjustments were made for potential confounders like gestational age, birth weight, gender, twin status, and Apgar score at 1 min. For results with few events or where predictor-outcome separation was nearly complete, Firth’s penalized likelihood approach was applied.

Statistical tests were two-tailed, and significance was set at *p* < 0.05.

## 3 Results

### 3.1 Infant/preterm care/management/treatment in infants with apnea of prematurity

**Figure 1:**
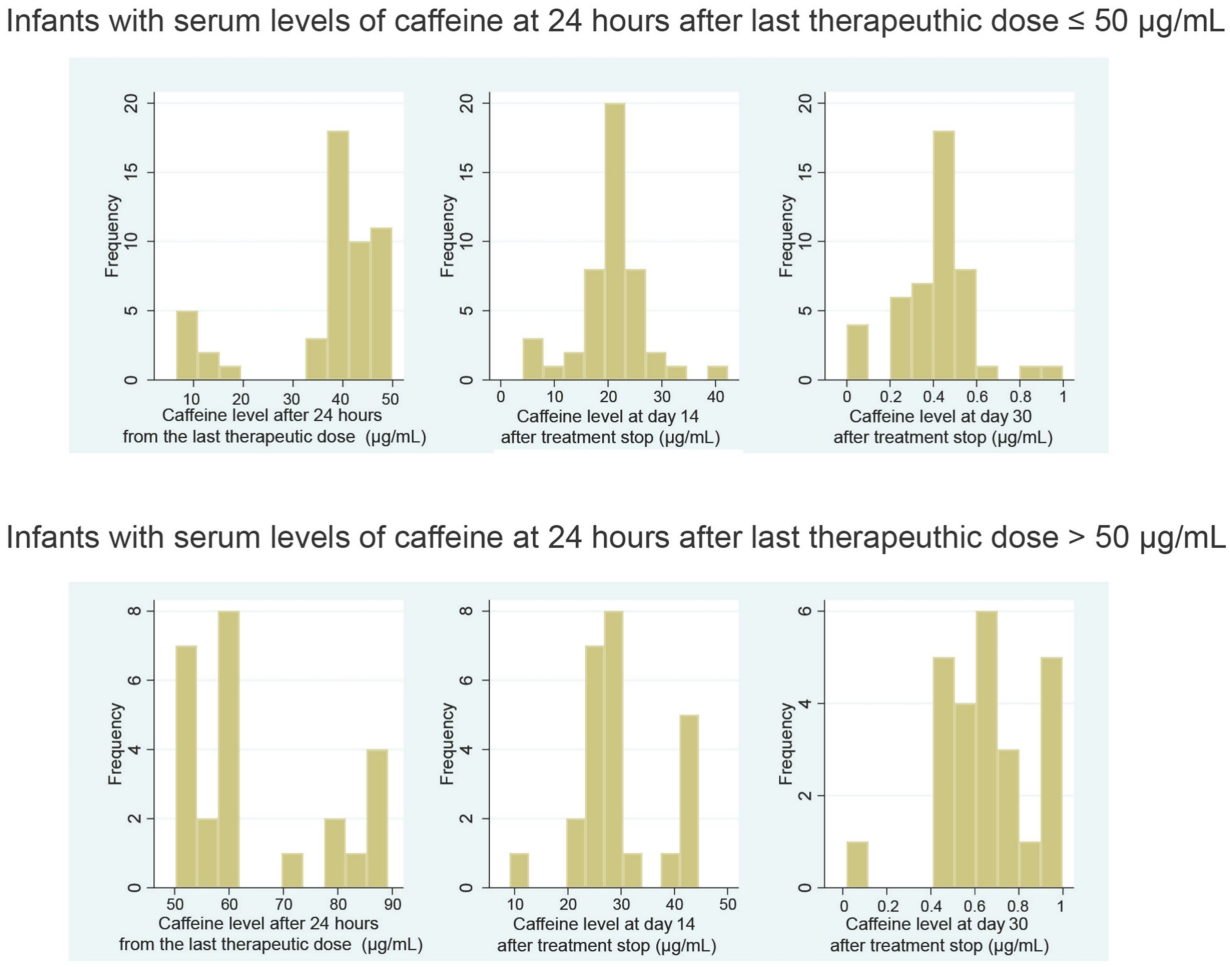
Serum caffeine concentration 24 h after the last therapeutic dose, 14 days after stopping treatment and 30 days after stopping treatment in infants with normal and high serum caffeine levels.

Premature infants given the standard dose of caffeine citrate demonstrated significant variability in serum caffeine concentrations 24 h after the final therapeutic dose, with levels comparable to values after the loading dose and significant concentrations exceeding 80 μg/mL in seven infants (Figure 1).

The results confirm the long-term persistence of caffeine citrate.

### 3.2 Comparison between neonates with normal versus high caffeine/xanthine levels

To further explore the potential impact of pharmacologic exposure, outcomes were compared between neonates with normal and elevated caffeine/xanthine levels (>50 μg/mL). Odds ratios were calculated after adjusting for gestational age, birthweight, gender, Apgar score at 1 min and twin status. This approach aimed to explore whether observed differences persisted after adjustment for key perinatal confounders, acknowledging that residual confounding by illness severity cannot be excluded.

To evaluate whether differences in respiratory support intensity confounded the association between high caffeine levels and neonatal complications, we compared respiratory interventions between neonates with normal and high caffeine/xanthine levels and included these variables in a sensitivity analysis (Table 1).

**Table 1:** Exploratory analysis of neonatal complications according to caffeine/xanthine levels (>50 μg/mL).

Variable	Normal caffeine levels	High caffeine levels	Adjusted OR	*p*
VPP neo-puff	86.00%	84.00%	0.85	0.817
No. of ventilations with Neo-Puff	21.86	25.24	-	0.120
nCPAP	22.00%	16.00%	0.68	0.540
Duration of nCPAP (hours)	52.36	72.00	-	0.053
Oro-tracheal intubation	42.00%	28.00%	0.54	0.237
Duration of intubation (hours)	2.38	2.57	-	0.502
Duration of free-flow O_2_ therapy (days)	5.34	7.52	-	0.017
Convulsive equivalents	22.72%	48.39%	3.18	0.020
Perinatal asphyxia	10.00%	0.00%	-	0.102
Respiratory complications	50.00%	60.00%	10.6	0.011
Intraventricular hemorrhage	4.00%	8.00%	3.45	0.156
Renal complications (acute)	2.00%	0.00%	-	0.476
Fluid/electrolyte imbalance	100.00%	100.00%	-	-
Ecchymosis	28.00%	36.00%	7.83	0.021
Edema	18.00%	24.00%	9.07	0.020
Allergic reactions	36.00%	36.00%	0.337	0.195
Hematologic complications	6.00%	0.00%	-	0.211
Vomiting	6.00%	0.00%	-	0.211
Heart murmur	0.00%	8.00%	-	0.043
Convulsions	6.00%	0.00%	-	0.211
Ankyloglossia	10.00%	20.00%	7.99	0.074
Hydrocele	8.00%	12.00%	0.45	0.521
Toe malposition	2.00%	0.00%	0	0.476
Partial syndactyly	2.00%	4.00%	2.04	0.612
Syndactyly agenesis	0.00%	8.00%	-	0.043
Congenital dermal melanocytosis	8.00%	8.00%	1	1.520
Short frenulum	10.00%	4.00%	0.38	0.367
Hemangioma	22.00%	24.00%	1.54	0.377
Favourable evolution	82.00%	80.00%	0.60	0.605
Lent favourable evolution	8.00%	20.00%	2.88	0.275
Severe evolution	10.00%	0.00%	0	0.102
Death	6.00%	0.00%	0	0.211

Odds ratio estimates for outcomes with cell counts <5 are statistically unstable and should be interpreted with caution.

Minor congenital anomalies are documented as part of the complete clinical record.

The use of Neo-Puff ventilation was similar between infants with normal and high caffeine levels (86.00% vs. 84.00%; adjusted OR = 0.85, *p* = 0.817), and the mean number of Neo-Puff inflations did not differ significantly (21.86 vs. 25.24; *p* = 0.120). Likewise, the proportion of infants requiring nCPAP was comparable (22.00% vs. 16.00%, adjusted OR = 0.68, *p* = 0.540), and orotracheal intubation rates did not differ between groups (42.00% vs. 28.00%, adjusted OR = 0.54, *p* = 0.237). The duration of intubation was similarly brief in both groups (2.38 vs. 2.57 h, *p* = 0.502).

However, the duration of nCPAP showed a trend toward longer use in the high caffeine group (72.00 vs. 52.36 h; *p* = 0.053), and the duration of free-flow O₂ therapy was significantly longer in infants with high caffeine levels (7.52 vs. 5.34 days; *p* = 0.017).

In the comparison between neonates with normal versus high caffeine/xanthine levels (>50 μg/mL), most complications showed no statistically significant difference between groups (Table 1). Respiratory complications and convulsive equivalents were more frequent in the high caffeine group with significant ORs (10.6 [*p* = 0.011] and 3.18 [*p* = 0.020]), respectively.

Some complications such as intraventricular hemorrhage, edema, and ankyloglossia showed higher rates in the high caffeine group. Adjusted ORs also showed that ecchymoses were associated with high caffeine levels (OR = 7.83, *p* = 0.021) and also with edema (OR = 9.07, p = 0.020). Other outcomes, such as heart murmur and syndactyly agenesis, were significantly more common in the high caffeine group, although the number of cases was low.

While the analysis suggested certain differences between neonates with normal and high caffeine/xanthine levels, most comparisons did not reach statistical significance, largely due to small event numbers.

Syndactyly agenesis (*p* = 0.043) and heart murmur (p = 0.043) appeared statistically significant, but each was based on only 1–2 events, rendering the *p*-values artefactual in the context of multiple comparisons and very limited statistical power.

In infants treated with very high doses of caffeine over 80 μg/mL due to severe hypoxia, hyponatremia was more frequent and they presented increased risk for intraventricular hemorrhage.

Model diagnostics for one edema demonstrated excellent discriminative ability (sensitivity = 73%, pseudo-R^2^ = 0.466, AUC = 0.896), with high specificity and overall correct classification, supporting the robustness of the high caffeine level association. The hemangioma model showed good discrimination (sensitivity = 47.1%, pseudo-*R*^2^ = 0.232, AUC = 0.819), though sensitivity was moderate consistent with the lower event rate in this subgroup.

### 3.3 Electrolytic and hydric imbalances at neonates with normal versus high caffeine levels

Sodium was significantly lower in infants with high serum caffeine (133.0 ± 3.7 mmol/L) vs. normal caffeine (138.6 ± 6.3 mmol/L), with a large effect size (Cohen’s d = 1.05) and *p* = 0.016. Potassium was lower in the high caffeine group (4.11 ± 0.68 vs. 4.57 ± 0.68 mmol/L), showing a moderate effect size (Cohen’s d = 0.676) but did not reach statistical significance (*p* = 0.093). Chloride was essentially identical between groups (104.93 vs. 104.92 mmol/L; d ≈ 0, *p* = 0.992). Calcium was also similar (1.19 vs. 1.18 mmol/L; small effect, *p* = 0.745) (Table 2).

**Table 2:** Electrolyte values (Na, K, Cl, Ca) and imbalances in preterm infants with normal and high serum caffeine levels.

		
	Normal caffeine (NC) (*n* = 50)	High caffeine (HC) (*n* = 25)	Cohen’s *d*/OR	*p*-value
Electrolyte serum values				
Na (mmol/L)	138.64 ± 6.34	133.01 ± 3.68	1.05	0.016
K (mmol/L)	4.57 ± 0.68	4.11 ± 0.68	0.676	0.093
Cl (mmol/L)	104.93 ± 3.15	104.92 ± 2.94	0.004	0.992
Ca (mmol/L)	1.19 ± 0.12	1.18 ± 0.0.10	0.132	0.745
Electrolyte imbalance				
Hypo Na	35.71%	72.73%	4.8	0.066
Hyper Na	7.14%	0%	0	0.366
Hypo K	6.67%	25.00%	4.67	0.183
Hyper K	33.33%	16.67%	0.4	0.326
Hypo Cl	0%	0%	–	–
Hyper Cl	28.57%	50.00%	2.5	0.263
Hypo Ca	46.15%	58.33%	1.63	0.543
Hyper Ca	15.38%	0%	0	0.157

Na, Sodium; K, Potassium; Cl, Chloride; Ca, Calcium.

Student t test was used to test the difference in means between the two groups. Chi^2^ test was used to test the difference of prevalence of electrolytic imbalances between the two groups.

Hyponatremia was more frequent in the high caffeine group (72.7% vs. 35.7%), with a large odds ratio (OR = 4.8) but borderline/non-significant (*p* = 0.066). The magnitude is clinically notable even if your sample is underpowered. Other imbalances (hypo−/hyper- potassemia, hyper-chloremia, hypo−/hyper-calcemia) showed directional differences, but none were statistically significant (all *p* > 0.15), consistent with limited power (n = 25 in the high caffeine group).

The data mainly support an association between high caffeine levels and lower sodium / more hypopotassemia, while other electrolytes look broadly similar.

### 3.4 Association between high_caffeine levels and antibiotic exposure

Table 3, presents the adjusted associations between high caffeine exposure (>50 μg/mL) and antibiotic exposure, accounting for gestational age, birth weight, sex, Apgar score at 1 min, and twin status. Generally, high caffeine exposure was associated with a higher intensity of antibiotic use, suggesting a relationship between high caffeine levels at 24 h after the last therapeutic dose and greater illness severity or increased clinical suspicion of infection.

**Table 3:** Association between high caffeine exposure and antibiotic exposure: odds ratios with 95% confidence intervals (adjusted for gestational age, birthweight, gender, Apgar score at 1 min and twin status).

Outcome	OR	95% CI	*p*
No. of antibiotics	2.34	1.09–5.04	0.030
Gentamycin	2.07	0.48–8.79	0.327
Meropenem	6.015	0.79–21.53	0.018
Ampicillin	0.54	0.14–2.03	0.360
Imipenem	1.84	0.23–14.88	0.568
Vancomycin	0.49	0.01–29.24	0.735
Amikacin	1.54	0.22–10.80	0.664
Ampicillin + Gentamycin	1.54	0.41–5.69	0.521
Meropenem + Gentamycin	2.80	0.77–10.21	0.118
Ampicillin + Gentamycin + Meropenem	4.11	0.79–21.53	0.094
Ampicillin + Gentamycin + Imipenem	1.84	0.23–14.88	0.568

OR, Odds Ratio; CI, Confidence Interval; OR/CI were exponentiated from coefficients.

High caffeine exposure was significantly associated with an increased number of antibiotics administered (OR = 2.34, *p* = 0.030), indicating that infants with elevated caffeine levels were more than twice as likely to receive multiple antimicrobial agents.

Among individual antibiotics, a statistically significant association was observed for meropenem (OR = 6.02, *p* = 0.018), suggesting that infants with high caffeine levels had markedly increased odds of receiving broad-spectrum therapy.

No statistically significant associations were identified for gentamicin, ampicillin, imipenem, vancomycin, or amikacin when analyzed individually, although point estimates for several agents were above unity, indicating a consistent trend toward increased exposure. Similarly, antibiotic combinations involving meropenem showed elevated odds ratios, particularly the triple combination of ampicillin, gentamicin, and meropenem (OR = 4.11), but these did not reach statistical significance.

### 3.5 Analysis of demographic and clinical-biological data in premature newborns without apnea of prematurity

In the exploratory analysis, the comparative group consisted of preterm infants without apnea of prematurity and was characterized by greater gestational maturity and overall physiological stability (Table 4). This group provides a reference profile for demographic, clinical, and paraclinical parameters in preterm neonates without respiratory instability.

**Table 4:** Clinical-biological parameters and demographic data in premature newborns in the comparison group (CoG).

Parameter	Mean ± standard deviation
Apgar score at 1 min	8.08 ± 0.71
Apgar score at 5 min	8.62 ± 0.49
Gestational age (weeks)	35.76 ± 0.49
Twin pregnancy	26.05%
Birth weight (kg)	2.47 ± 0.45
Male gender (%)	45.38%
Urban residence (%)	28.99%
VPP Neo-Puff	94.96%
No. of ventilations with Neo-Puff	15.86 ± 8.69
nCPAP	17.65%
Duration of nCPAP (hours)	45.33 ± 38.46
Oro-tracheal intubation	7.56%
Duration of intubation (hours)	38.67 ± 12.04
Duration of Fio2 (days)	2.59 ± 0.74
Oxygen saturation (SO₂, %)	91.11 ± 8.20
PaCO₂ (mmHg)	38.22 ± 9.92
pH (arterial)	7.37 ± 0.07
PaO₂ (mmHg)	59.06 ± 17.51
Carboxyhemoglobin (%)	0.76 ± 0.43
Methemoglobin (%)	0.45 ± 0.23
Oxyhemoglobin fraction (%)	90.30 ± 8.37
HCO_3_ (mmol/L)	21.58 ± 2.66
Total bilirubin (mg/dL)	13.97 ± 3.75
Direct bilirubin (mg/dL)	0.52 ± 0.44
Glucose (mg/dL)	63.31 ± 19.55
Lactic acid (mmol/L)	2.99 ± 2.08
Total protein (g/dL)	4.93 ± 0.61
C reactive protein (mg/dL)	0.75 ± 1.55
Interleukin-6 (pg/mL)	24.13 ± 43.96
TNF alpha (pg/mL)	7.47 ± 4.46
Procalcitonin (ng/mL)	11.95 ± 17.96
Hemoglobin (g/dL)	15.27 ± 2.66
Hematocrit (%)	42.72 ± 7.48
Red blood cells (10^6/μL)	4.10 ± 0.69
MCV (fL)	104.03 ± 4.99
MCH (pg)	37.22 ± 1.74
MCH concentration (g/dL)	35.77 ± 0.80
White blood cells (10^3/μL)	16.59 ± 5.72
Neutrophils (%)	65.04 ± 10.09
Eosinophils (%)	1.35 ± 1.27
Basophils (%)	0.13 ± 0.10
Lymphocytes (%)	25.11 ± 6.59
Monocytes (%)	7.44 ± 1.96
Platelets (10^3/μL)	293.89 ± 143.60
Erythroblasts (per 100 leucocytes)	2.43 ± 1.17

TNF, Tumor Necrosis Factor; IL, Interleukin; PCT, Procalcitonin; CRP, C-Reactive Protein; MCV, Mean Corpuscular Volume; MCH, Mean Corpuscular Hemoglobin; MCHC, Mean Corpuscular Hemoglobin Concentration.

The comparative group had a median gestational age of 36 weeks and a median birth weight of 2.47 kg, reflecting a more advanced stage of intrauterine development. Early postnatal adaptation was favorable, with mean Apgar scores of 8.08 at 1 min and 8.62 at 5 min, indicating effective cardiopulmonary transition at birth. Male infants accounted for 45.38% of the comparative group. Most neonates originated from rural areas (71.01%), and singleton pregnancies predominated, with twin gestations accounting for 26.05% of cases.

Respiratory and gas exchange parameters were within stable ranges. Mean oxygen saturation was 91.11%, and oxyhemoglobin fraction averaged 90.3%, indicating adequate oxygenation. Arterial blood gas analysis showed a mean PaO₂ of 59.06 mmHg, PaCO₂ of 38.22 mmHg, and arterial pH of 7.37, consistent with effective ventilatory control. Levels of carboxyhemoglobin and methemoglobin remained low, suggesting minimal interference with oxygen delivery.

Biochemical assessment demonstrated preserved metabolic balance. Total serum proteins averaged 4.93 g/dL, supporting adequate nutritional and hepatic function. Total bilirubin levels averaged 13.97 mg/dL, remaining within expected limits for preterm neonates in the early postnatal period. Markers of inflammation were generally low in the comparative group. C-reactive protein levels were minimal, and TNF-*α* concentrations averaged 7.47 pg./mL, indicating the absence of significant systemic inflammatory activation.

Hematological evaluation revealed stable complete blood count parameters. White blood cell and neutrophil counts were within expected neonatal ranges, with balanced lymphocyte, monocyte, and eosinophil fractions. Erythroblast counts were low, reflecting physiologic erythropoiesis without evidence of hematologic stress (Table 4).

By contrast, in the AOP group mean gestational age of 33.23 weeks and birthweight was 2.01 kg, lower than in comparative group, while in AOP group there were more male infants (62.67%) and most infants were from urban areas (61.33%). In AOP group the gas exchange and acid–base parameters were lower than in comparative group (oxygen saturation 86.02%, CO_2_ partial pressure 40.46 mmHg and pH 7.34).

It should be noted that the difference in gestational age between the AOP and comparative groups is the primary expected determinant of AOP occurrence, and all observed inter-group differences must be interpreted in this context. This analysis is therefore descriptive and serves only to characterize the two cohorts; it does not permit causal or etiological inference.

### 3.6 Neonatal complications in infants with and without apnea of prematurity

We further assessed the need for respiratory interventions and frequency of neonatal complications in infants with apnea of prematurity in the exploratory analysis (Table 5). Particular attention was paid to orotracheal intubation practices, duration of oxygen therapy, spectrum of comorbidities and associated outcomes, to capture the broader clinical impact of apnea of prematurity. The following analysis is presented for descriptive purposes only, to characterize the clinical profile of the AOP cohort relative to gestational-age-matched premature infants without apnea. It is not intended to establish etiological or causal associations, as the two groups differ fundamentally in gestational age, which is the primary determinant of AOP frequency.

**Table 5:** Neonatal complications in infants with apnea of prematurity versus comparison group.

Variable	Comparison group CoG Median [IQR]) (%)	Apnea of prematurity group (HC, NC) Median [IQR] (%)	OR/variation (%)	*p*-value
VPP Neo-Puff	94.96%	83.33%	0.31	0.005
Number of Neo-Puff inflations	10 (10–20)	25 (15–30)	+150%	<0.001
nCPAP	17.65%	20.00%	1.17	0.645
Duration of nCPAP (hours)	72 (1–84)	72 (48–72)	+0.0%	0.499
Oro-tracheal intubation	7.56%	14.70%	7.28	<0.001
Duration of intubation (hours)	48 (24–48)	2 (2–3)	−95.8%	<0.001
Duration FiO₂ therapy (days)	3 (2–3)	5 (4–8)	+66.7%	<0.001
Convulsive equivalents	14.80%	41.33%	4.06	<0.001
Perinatal asphyxia	0.00%	6.67%	–	<0.001
Respiratory complications	5.04%	53.33%	21.52	<0.001
Intraventricular hemorrhage	0.00%	5.33%	–	<0.001
Renal complications (acute)	0.00%	1.33%	–	0.074
Fluid/electrolyte imbalance	35.71%	100.00%	–	<0.001
Ecchymosis	1.60%	30.67%	2.33	0.005
Edema	18.07%	20.00%	1.13	0.707
Allergic reactions	34.45%	36.00%	1.07	0.806
Vomiting	2.94%	4.00%	1.38	0.649
Jaundice	100.00%	100.00%	–	1.000
Heart murmur	0.00%	2.67%	–	0.011
Convulsives	0%	4.00%	–	0.019
Ankyloglossia	0.00%	13.33%	–	<0.001
Hydrocele	0.00%	9.33%	–	<0.001
Toe malposition	0.00%	1.33%	–	0.074
Partial syndactyly	0.00%	2.67%	–	0.011
Syndactyly agenesis	0.00%	2.67%	–	0.011
Congenital dermal melanocytosis	7.98%	8.00%	1	0.996
Short frenulum	2.94%	8.00%	2.87	0.055
Hemangioma	60.50%	22.67%	0.19	<0.001
Favourable evolution	92.86%	81.33%	0.34	0.004
Lent favourable evolution	7.14%	12.00%	1.77	0.184
Severe evolution	0.00%	6.67%	–	<0.001
Death	0.00%	4.00%	–	0.019

IQR, interquartile range; OR, odds ratio.

In the group with apnea of prematurity, orotracheal intubation was notably more common than in the comparison group (14.7% vs. 7.6%; *p* < 0.001), due to the need for surfactant administration, although the median duration of intubation in these infants was dramatically shorter (2 vs. 48 h; p < 0.001). Additionally, the apnea of prematurity group required extended periods of oxygen titrated in the blender (5 vs. 3 days; *p* < 0.001).

These infants also experienced a significantly higher burden of neonatal complications. Perinatal asphyxia and respiratory complications were markedly elevated (6.67% vs. 0 and 53.33% vs. 5.0%; both p < 0.001), as were incidences of intraventricular hemorrhage (5.33% vs. 0%), fluid and electrolyte imbalances (100% vs. 35.7%; both p < 0.001), convulsive equivalents (41.33% vs. 14.80%; p < 0.001) and ecchymosis (30.67% vs. 1.60%; *p* = 0.014). Hematologic complications, heart murmurs, ankyloglossia, hydrocele, partial syndactyly, syndactyly agenesis, severe outcomes, and mortality were all significantly more frequent in the apnea of prematurity group versus comparison group (all *p* < 0.05). Finally, clinical outcomes were less favorable in infants with apnea of prematurity: the proportion achieving favorable outcomes was lower (81.33% vs. 92.86%; *p* = 0.004), while severe outcomes and death were more common (4.00% vs. 0%; *p* = 0.019).

During the study period, three preterm infants in the apnea of prematurity group (4.00%) died, while no deaths occurred in the comparison group (p = 0.019). The causes of death were as follows: Case 1 died from severe intraventricular hemorrhage (Grade III) with subsequent multiorgan failure; Case 2 died from septic shock with disseminated intravascular coagulation; Case 3 died from progressive respiratory failure complicated by enterocolitis and cardiovascular collapse. All three newborns survived 6, 17 and 23 h, respectively, during which they were administered only the loading dose of caffeine citrate, therefore they belong to the NC group with caffeine level <50 μg/mL. The evolutionary state was extremely serious, they developed refractory hypotension, seizures, multiorgan dysfunction and cardiorespiratory arrest, which did not respond to advanced resuscitation measures.

Cranial ultrasonography revealed intraventricular hemorrhage (IVH) in four patients from the group with apnea of prematurity (5.33%), attributed to the immaturity of the blood–brain barrier. According to the Papile classification system (24), the severity distribution was as follows: Grade I (germinal matrix hemorrhage only) in 2 patients (50%), Grade II (intraventricular hemorrhage without ventricular dilatation) in 1 patient (25%), and Grade III (intraventricular hemorrhage with ventricular dilatation) in 1 patient (25%). No Grade IV hemorrhages (IVH with parenchymal involvement) were observed.

## 4 Discussion

Prematurity remains a major public health concern, frequently associated with neurological impairments that hinder psychomotor development. This study identified distinct clinical, respiratory, hematologic, and inflammatory differences between preterm infants with and without apnea of prematurity. The findings highlight the multifactorial nature of apnea, reflecting contributions from developmental immaturity, altered gas exchange, and immune dysregulation. These results align with prior evidence on the vulnerability of preterm populations and provide additional insights into the systemic mechanisms underlying apnea of prematurity. Due to the difference in gestational age between the AOP and comparative groups all observed inter-group differences are therefore descriptive and serve only to characterize the two cohorts.

Current research indicates that caffeine is widely used in preterm infants to stimulate the cardiorespiratory system, reduce apnea, and improve respiratory outcomes, with standard dosing regimens generally considered safe and effective for most infants (18). Caffeine therapy has been shown to increase respiratory effort, reduce the incidence of intermittent hypoxemia, and decrease the need for mechanical ventilation and the duration of noninvasive respiratory support, without significant adverse effects at standard doses (25).

The effect of methylxanthines can be either potentiated or inhibited when administered concomitantly with other drugs, and variations in serum concentration can be associated with adverse reactions such as fever, tachypnea, fasciculations, vomiting, hyperglycemia, hypokalemia, uremia, allergic reactions, leukocytosis, anemia, necrotizing enterocolitis, and seizures (16, 18). The focus was not on the beneficial therapeutic effects of methylxanthines, but rather on the associations of elevated caffeine levels with clinical manifestations, depending on patient compliance.

The dynamic frequency of complications is much higher in the apnea of prematurity group versus comparison group. For this reason, selective monitoring of plasma methylxanthine concentrations may prevent the occurrence of side effects at caffeine titers higher than 50 μg/mL.

In our study, premature infants receiving caffeine citrate demonstrated marked interindividual variability in plasma caffeine levels, with some values exceeding 80 μg/mL. Serum levels detected 14 days after the last maintenance dose demonstrated slow caffeine metabolism and prolonged persistence of the therapeutic effect after discontinuation, highlighting that gradual dose reduction is unnecessary at treatment cessation Because it is often difficult to distinguish between common complications of prematurity and secondary reactions to methylxanthines, serum monitoring of plasma drug concentrations may maintain optimal levels consistent with the patient’s clinical condition. Although caffeine appears to be better tolerated than theophylline (26–31), selective dosing of caffeine citrate is necessary to ensure that plasma caffeine levels remain below 50 μg/mL, thereby minimizing the risk of adverse effects associated with this respiratory analeptic.

In real plasma samples, as reported in the literature, methylxanthine levels demonstrated wide interindividual variability. This variability reflects complex factors such as differences in absorption and metabolism rates, co-administration with nutrition, type of milk, and concurrent medications, as well as hepatic immaturity, enzyme inhibition or induction, and treatment compliance (32). No universally accepted therapeutic serum interval has been established. In preterm infants, the prolonged plasma half-life of methylxanthines predisposes to accumulation at high concentrations, above the critical threshold.

Our findings support the sequential administration of a loading dose and the first two maintenance doses, followed by gradual adjustment of caffeine citrate intake according to measured plasma caffeine levels, without exceeding 50 μg/mL. The markedly reduced metabolism of caffeine in preterm newborns is attributable to the immaturity of hepatic enzyme systems, with most active substances being eliminated renally (33). In addition, placental histopathological abnormalities particularly villous immaturity, vascular thrombosis, fibrinoid deposition, and evidence of chronic hypoperfusion reflect impaired fetoplacental circulation and intermittent fetal hypoxia. These intrauterine conditions are known to influence the maturation of fetal organs, including the liver. In preterm neonates, hepatic enzymatic systems responsible for caffeine metabolism (primarily cytochrome P450 isoenzymes, especially CYP1A2) are already immature; however, chronic intrauterine hypoxia and placental insufficiency may further delay hepatic functional development. Reduced hepatic perfusion and oxygenation can impair enzyme activity, while systemic inflammation associated with placental pathology may additionally downregulate metabolic pathways.

Consequently, neonates originating from structurally and functionally compromised placentas may exhibit reduced caffeine clearance, prolonged half-life, and increased susceptibility to drug accumulation.

Given the risk of apnea recurrence after discontinuation of caffeine citrate treatment, dynamic monitoring of plasma titer could prevent this complication, although low plasma caffeine concentrations have been detected in some infants even 1 month after cessation of therapy.

Caffeine pharmacokinetics in preterm neonates are highly variable, with prolonged half-life and dependence on developmental maturation and illness severity (34). Consequently, high serum caffeine levels may represent both increased pharmacological exposure and reduced metabolic clearance. In either case, their co-occurrence with electrolyte disturbances likely reflects shared underlying physiological vulnerability rather than a simple causal chain from caffeine to electrolyte imbalance (16).

We examined next the relationship between high caffeine exposure (>50 μg/mL) and neonatal complications. We observed some differences between groups (HC and NC), except for rare anomalies such as syndactyly agenesis and partial syndactyly These findings suggest that elevated serum caffeine levels are associated with selected neonatal outcomes; however, given the observational design and small event counts, they should be considered hypothesis-generating only.

The effects of supratherapeutic caffeine exposure are not clear in the literature. While higher doses may further reduce bronchopulmonary dysplasia, observational studies and some trials suggest that excessive dosing could be associated with poor tolerability and potential harm, especially in the most immature infants, and there is a need for caution due to the risk of side effects and possible complications such as intracranial bleeding (25, 35). The optimal dosing and timing of caffeine therapy remain unresolved, and more research is needed to determine the safety and efficacy of high-dose regimens, particularly regarding long-term neurodevelopmental outcomes and cardiorespiratory stability (35).

The landmark Caffeine for Apnea of Prematurity (CAP) trial demonstrated that administering caffeine at a loading dose of 20 mg/kg followed by a daily maintenance dose of 5–10 mg/kg in infants weighing less than 1,251 g, either extubated from mechanical ventilation or receiving therapy for apnea, significantly reduced the requirement for respiratory support. This reduction was associated with a decreased incidence of bronchopulmonary dysplasia, a chronic lung disease that frequently complicates the course of extremely preterm infants, thereby improving overall pulmonary health in early life. Moreover, long-term follow-up assessments extended into childhood demonstrated not only better neurodevelopmental outcomes, such as improved motor and cognitive performance, but also suggested a sustained benefit in growth and functional capacity compared with peers who did not receive caffeine therapy (16).

Current research does not support improved neurodevelopmental outcomes in preterm infants managed with higher doses of caffeine compared to the standard dose (20 mg/kg loading, 5 mg/kg maintenance) (36). A pilot randomized trial found that a high loading dose (80 mg/kg) led to a higher incidence of cerebellar hemorrhage and more abnormal neurologic signs at term equivalent age (17), with no difference in neurodevelopmental outcomes at 2 or 5 years compared to the standard dose (37). Similarly, a recent systematic review and meta-analysis concluded that while higher caffeine doses may be more effective for reducing apnea and bronchopulmonary dysplasia, they do not improve neurocognitive outcomes in early childhood, and the optimal dose for neurodevelopment remains unknown (38).

Multidisciplinary collaboration represents the most important aspect in the care of preterm newborns regarding continuous monitoring, since these patients can rapidly develop various complications. The antenatal administration of glucocorticoids to pregnant individuals, when preterm birth is anticipated, can prevent or mitigate neonatal respiratory distress syndrome (39, 40). The onset of hypothermia was counteracted by promptly wrapping the body and unexposed extremities in plastic sheets, and resuscitation was performed on a radiant heated table (41).

All preterm newborns included in the study required initial respiratory support due to pulmonary immaturity and limited respiratory muscle strength. Support was provided by administering titrated oxygen to the blender or with the aid of the Neopuff device (a T-piece resuscitator that delivers appropriate positive end-expiratory pressure and controlled inflation pressures). The main mode of ventilation was pressure-limited conventional ventilation of the SIMV (synchronized intermittent mandatory ventilation) type. The lowest tidal volume ensuring adequate oxygenation and a short inspiratory time were used. Limiting hypoxia-hyperoxia fluctuations helps prevent chronic lung disease and the incidence of retinopathy of prematurity. Newborns with RDS received the first dose of surfactant as soon as possible after intubation to allow rapid transition to continuous positive airway pressure (CPAP) support shortly after administration. The therapeutic strategy aimed to maintain and establish functional residual capacity through the administration of surfactant and continuous positive airway pressure (42). In cases of inefficient respiratory effort and apnea, we employed intermittent positive-pressure ventilation and sustained nasal CPAP for a period of time (43).

The findings highlight the central role of respiratory support in the management of apnea of prematurity. Infants with apnea of prematurity demonstrated greater reliance on invasive and non-invasive interventions, including higher rates of intubation and prolonged oxygen therapy.

Our study identified an association between high serum caffeine levels and respiratory complications in AOP infants, consistent with existing literature on the narrow therapeutic margin of caffeine (18). Whether supratherapeutic exposure directly destabilizes cardiorespiratory control, or alternatively reflects greater underlying physiological instability leading to reduced caffeine clearance, cannot be determined from observational studies.

In response to the potential confounding effect of illness severity on the association between elevated caffeine levels and neonatal outcomes, we examined respiratory support intensity as a proxy for clinical acuity. The use and frequency of Neo-Puff ventilation, the proportion of infants requiring nCPAP or orotracheal intubation, and the duration of intubation were all comparable between the normal and high caffeine groups, indicating that the two cohorts received broadly similar levels of acute respiratory intervention. The duration of intubation was notably brief in both groups (approximately 2.4–2.6 h), reflecting the institutional practice of early surfactant administration followed by rapid extubation to non-invasive support.

High serum caffeine levels were associated with significantly lower sodium concentrations and a markedly higher prevalence of hyponatremia, with a large effect size. Although the difference in hyponatremia prevalence narrowly missed statistical significance, the magnitude of the odds ratio suggests potential clinical relevance and may reflect limited statistical power in the high-caffeine group. This association is biologically plausible. Caffeine, as a non-selective adenosine receptor antagonist, blocks renal adenosine A1 receptors, which normally promote sodium reabsorption in the proximal tubule and mediate tubuloglomerular feedback. Blockade of these receptors produces natriuretic and diuretic effects, increasing urinary sodium excretion. In preterm infants, immature renal tubular function and reduced urinary concentrating capacity amplify these effects, particularly at supratherapeutic caffeine concentrations (22).

Hyponatremia in preterm neonates can worsen neurological instability, including seizure threshold reduction and may compound the respiratory dysregulation already present in AOP, creating a pathophysiological feedback loop.

Potassium levels showed a non-significant trend toward lower values in the high caffeine group, while chloride and calcium concentrations were comparable between groups, suggesting that caffeine exposure may preferentially affect sodium homeostasis rather than causing a generalized electrolyte imbalance.

Gas exchange and acid–base balance findings support the concurrent monitoring of serum electrolytes, particularly sodium, in preterm infants with elevated caffeine levels and warrant further investigation in larger, prospective studies to clarify causality and dose–response relationships.

In the present study, high caffeine exposure was associated with a greater burden of antibiotic therapy, even after adjustment for key perinatal confounders. Infants with high caffeine levels had significantly higher odds of receiving a greater number of antibiotics, as well as a markedly increased likelihood of exposure to meropenem, a broad-spectrum agent typically reserved for severe or refractory infections. Although most individual antibiotics and combination regimens did not reach statistical significance, the consistently elevated odds ratios suggest a trend toward more intensive antimicrobial use in this subgroup.

These findings likely reflect increased clinical severity, instability, or heightened suspicion of infection in infants with elevated caffeine levels, rather than a direct causal effect of caffeine on infection risk (44). However, given the known pharmacokinetic variability of caffeine in premature neonates and its potential systemic effects at high concentrations it is also possible that certain manifestations associated with supratherapeutic caffeine concentrations — such as tachycardia, irritability, or metabolic disturbance — may overlap clinically with signs of infection, potentially contributing to increased diagnostic uncertainty (45) and sepsis can influence the antibiotic concentrations. However, this mechanism is speculative in the absence of individual-level data on the sequence of caffeine elevation and antibiotic initiation.

Morphological examination of the blood provided numerous valuable insights into complications associated with prematurity, such as anemia, thrombocytopenia (46), the intensity of hemolysis evidenced by the presence of erythroblasts, anisocytosis and poikilocytosis of erythrocytes, and a left shift in the leukocyte formula during infections. Therapy with Neorecormon, combined with adequate iron supplementation, resulted in accelerated erythropoiesis but could not replace erythrocyte transfusions in cases of significant blood loss or severe hemolytic processes (47). Dynamic assessment of leukocyte counts and differentiated blood smears aided in evaluating the risk of maternal-fetal infection, enabling a thorough analysis of the impact of gestational age differences, complications arising at birth, and postnatal age.

Lower total leukocyte and neutrophil counts in the apnea of prematurity cohort are consistent with well-described innate immune immaturity in preterm infants, including reduced neutrophil production and impaired chemotaxis, phagocytosis, and oxidative burst, features that can manifest as quantitative neutropenia in the absence of infection (48). The relative increase in lymphocytes aligns with age-specific differentials and reference intervals showing higher lymphocyte proportions in early neonatal life, particularly in very preterm infants (49). Elevated eosinophil and monocyte percentages are also compatible with neonatal hematologic patterns: eosinophilia is more common in smaller preterms and in NICU contexts with multiple stressors (50), while monocyte number and function show developmental differences, with skewed cytokine responses, compared with term infants (51).

Finally, the higher erythroblast (nucleated red blood cell) counts support heightened erythropoietic activity, a recognized response to intrauterine or postnatal hypoxemia and stress that correlates with adverse neonatal conditions (52).

Preterm newborns require long-term medical care to monitor, promptly identify, and manage respiratory and neurological deficiencies, as well as to evaluate potential cardiovascular dysfunctions and the onset of auditory and visual dysfunction. Children born prematurely face an elevated risk of disabilities, necessitating early supplementary screening and multiple subsequent evaluations of neurological development.

The mortality rate in the apnea group (4.00%) was significantly higher than in comparison group (0%, *p* = 0.019), reflecting the increased vulnerability of infants with apnea of prematurity to life-threatening complications. All deaths occurred in extremely preterm infants with very low birth weight and were attributed to multisystem organ failure rather than isolated apnea events. These findings align with established literature demonstrating that apnea of prematurity serves as a marker of overall physiological immaturity and systemic vulnerability, with mortality primarily driven by associated complications rather than apnea *per se* (14, 22).

The intraventricular hemorrhages were attributed to hypoxic–ischemic injury and/or sudden changes in cerebral blood volume associated with immature cerebrovascular autoregulation and fragility of the germinal matrix vasculature, as detected by transfontanellar ultrasound examination. The absence of Grade IV hemorrhage (parenchymal involvement) in this cohort may reflect timely respiratory support and careful hemodynamic management, though the presence of Grade III hemorrhage underscores the vulnerability of extremely preterm infants to severe neurological complications (53, 54).

Bronchopulmonary dysplasia (BPD), or chronic lung disease of prematurity - as defined by the 2001 National Institutes of Health conference - affects premature infants with extremely low birth weight due to inflammation and lung injury induced by oxygen administration and barotrauma caused by mechanical ventilation, which in turn arrest lung development and reduce the surface area for gas exchange. BPD therapy includes bronchodilators, glucocorticoids, and diuretics, though there are currently no strategies supported by robust evidence (55).

The substantially higher rates of orotracheal intubation in preterm infants with apnea likely reflect the need for immediate airway support in the context of unstable respiratory control. However, the brevity of intubation suggests that clinicians prioritized early transition to non-invasive respiratory modalities, consistent with current NICU practices emphasizing minimal mechanical ventilation to reduce lung injury and long-term morbidities like bronchopulmonary dysplasia (36). Prolonged reliance on oxygen titrated in the blender further underscores the underlying pulmonary and respiratory control immaturity in apnea of prematurity (56). These findings align with consensus guidelines affirming apnea of prematurity as a manifestation of underdeveloped ventilatory regulation predisposes infants to frequent hypoxemic episodes necessitating long time support (36).

These patterns reflect the underlying immaturity of respiratory control in preterm infants (57) and underscore the need for careful balancing between stabilization and minimizing the risks associated with prolonged mechanical ventilation.

The apnea of prematurity group bore a substantially higher burden of neonatal complications, including perinatal asphyxia and respiratory issues, intraventricular hemorrhage, fluid and electrolyte imbalances, and a range of other systemic and anatomical complications. Clinical outcomes were comparatively poorer, with fewer achieving favorable results and higher rates of severe outcomes and mortality.

The high prevalence of multisystem complications – including respiratory morbidities, intraventricular hemorrhage, and pronounced fluid and electrolyte disturbances – highlights the systemic vulnerability of the apnea of prematurity group. In particular, electrolyte imbalance has been recognized as a contributor to neurologic and respiratory instability in preterm infants, often correlating with compounded risk for adverse outcomes (56).

The higher prevalence of minor congenital findings in the AOP cohort is most likely attributable to the lower gestational age and development disturbances of this group rather than reflecting a specific biological relationship with apnea of prematurity. But in our study we had a low number of occurrences of these conditions and therefore a low statistical power. Their association with high caffeine levels can be due to decreased metabolism of caffeine in premature liver. Increased rates of severe clinical outcomes and mortality in the AOP group reinforces apnea of prematurity as a marker of increased hypoxic risk (57) These observations mirror established clinical literature linking apnea-related hypoxic and bradycardic events to long-term neurodevelopmental sequelae and other systemic pathologies (56, 57).

While the observational nature of this study precludes definitive causal conclusions regarding the role of supratherapeutic caffeine exposure in neonatal outcomes, the pattern of associations observed is consistent with a need for individualized TDM-guided caffeine dosing. Collectively, these results support the need for vigilant, multidisciplinary management approaches tailored to infants with apnea of prematurity, focusing on early respiratory support, careful monitoring of metabolic and hemodynamic status, and minimizing invasive ventilation to improve both short- and long-term outcomes. Increasing glucose availability in the hippocampus after seizure onset may reduce seizure severity, decrease neuronal cell death, and support neuroprotection, likely through upregulation of glucose and sodium cotransporter 1 (SGLT1) (58).

Gestational age and birth weight are consistent with immaturity of respiratory control inversely proportional to maturity, placing immature newborns at greater risk of developing apnea of prematurity, findings supported by predictive modeling studies in preterm populations (59). The male preponderance in AOP group aligns with established increased vulnerability in male preterms. Higher representation from urban areas in AOP group may reflect increased referral patterns or environmental exposures, meriting further exploration.

These results underscore the importance of therapeutic drug monitoring and individualized caffeine dosing in preterm infants, not only to reduce direct adverse effects, but also to potentially limit unnecessary exposure to broad-spectrum antibiotics and their associated risks (44, 60–63).

Preterm infants with apnea demonstrate evidence of perinatal instability, impaired respiratory physiology, and altered inflammatory/hematologic/coagulation states (46). These differences likely reflect a confluence of immaturity across multiple systems, predisposing them to apnea. Recognizing these patterns early could improve stratification and individualized care in the NICU.

### 4.1 Limitations

This research is not without limitations. Potential cross-reactions and interferences may be due to hemolyzed, lipemic icteric biological samples, the potentiating or inhibitory effect of concomitant medications administered during prematurity therapy, or maternal consumption of xanthine-containing foods and beverages during pregnancy and lactation.

The perfect correlation values and large ORs observed in some analyses may reflect limited variability in the data and highlight the need for validation in larger cohorts, as our sample size was relatively small (*n* = 25). The present study involved exploratory multivariable logistic regression across multiple clinical outcomes, which increases the risk of false-positive findings due to type I error inflation. Although model diagnostics were satisfactory for the primary outcomes of interest, notably neonatal edema and hemangioma, several secondary outcomes exhibited complete or quasi-complete separation, precluding reliable coefficient estimation.

Also, the study design was prospective observational, precluding inferences about causality or directionality, and the reported associations should be interpreted as correlative. Therefore the study does not permit discrimination between high caffeine acting as a contributor to clinical presentations that mimic infection, and infection-related metabolic alterations causing caffeine accumulation.

High serum caffeine concentrations in our cohort may represent a surrogate marker of greater physiological vulnerability — reflecting immature or compromised drug metabolism rather than a direct pharmacotoxic effect. Confounding by illness severity cannot be excluded in this observational setting, and the associations reported should be interpreted as hypothesis-generating only.

Individual-level data on the temporal sequence of caffeine elevation relative to sepsis onset and antibiotic initiation were not systematically recorded. The observational design does not permit discrimination between high caffeine acting as a contributor to clinical presentations that mimic infection, and infection-related metabolic alterations (e.g., hepatic hypoperfusion, cytokine-mediated CYP1A2 downregulation) causing caffeine accumulation.

Certain parameters – such as caffeine levels – were available only for the apnea of prematurity group, which restricted comparative analyses. In addition, the utility of Apgar scoring in extremely preterm populations remains debated, warranting cautious interpretation. Finally, some complications identified (e.g., congenital anomalies) may overlap with prematurity-related risks rather than being specific to apnea of prematurity.

### 4.2 Future directions

The results of this study highlight several opportunities for translation into neonatal intensive care practice. Clinicians should consider early interventions, such as serum methylxanthine monitoring, prophylactic noninvasive respiratory support, aggressive correction of electrolyte imbalances, targeted metabolic stabilization, and kangaroo care, as part of a comprehensive strategy to reduce complication rates and improve long-term outcomes. Particular attention should be paid to proactive management of anemia and electrolyte disturbances, given their strong associations with seizures, edema, and systemic instability.

In addition to individual interventions, the integration of predictive tools that associate gestational age, laboratory biomarkers, and perinatal characteristics into clinical decision-making may allow for more precise tailoring of therapies and improve evolutionary risk stratification. These approaches may be beneficial in the efficient allocation of resources in neonatal intensive care units, while facilitating medical safety and quality of care for vulnerable preterm infants.

Overall, more robust studies could advocate for the inclusion of therapeutic drug monitoring and personalized methylxanthine dosing strategies in national and international neonatal care guidelines, thus ensuring that precision medicine principles are integrated into clinical practice.

## 5 Conclusion

Correlating the clinical-pathological evolutionary data of premature patients constitutes objective support for the opportunity to optimize the doses of methylxanthines administered to treat and prevent episodes of apnea of prematurity depending on the serum caffeine citrate titer, oxygenation index and clinical status. Implementing individualized methylxanthine therapy guided by therapeutic drug monitoring (TDM) can reduce adverse effects, enhance respiratory stability, and support safer neonatal care.

Personalized pharmacological interventions in apnea of prematurity may serve as a model for precision medicine approaches in neonatal intensive care units. Consequently, analysis of serum methylxanthine titers in critical cases is necessary to guarantee therapeutic effects exclusively for the benefit of the patient.

## Data Availability

The original contributions presented in the study are included in the article/Supplementary material, further inquiries can be directed to the corresponding author.
