# Supplementary material for: Clinical and biological impact of caffeine therapy in premature infants with apnea of prematurity: a prospective observational study
Source: Front Med (Lausanne). 2026 May 13;13:1808401. doi: 10.3389/fmed.2026.1808401 (PMC13212477; doi:10.3389/fmed.2026.1808401)
Supplement: Supplementary file 1 [file Supplementary_file_1.docx]

Supplementary material

3.3. Histopathological findings in infants with and without apnea of prematurity

Microscopic examination of placental samples from 25–37 weeks gestation showed notable variability in chorionic villi size (Figures 2 and 3), reduced or absent cellularity within the villous axis, and stromal laxity (figures 2 and 3). Some vascular structures exhibited endothelial discontinuities and pseudoangiomatous features (figures 4 and 5). Immature intermediate villi predominated (Figure 6), and a well-formed cytotrophoblast with reduced syncytiotrophoblast indicated placental immaturity. Peri- and intervillous fibrinoid deposits with vascular thrombosis were present (figures 7 and 8), along with necrotic and hemorrhagic lesions (Figure 9), calcific and microhemorrhagic foci (Figure 10), and macrophages containing hemosiderin (Figure 11), collectively reflecting features of premature placenta.


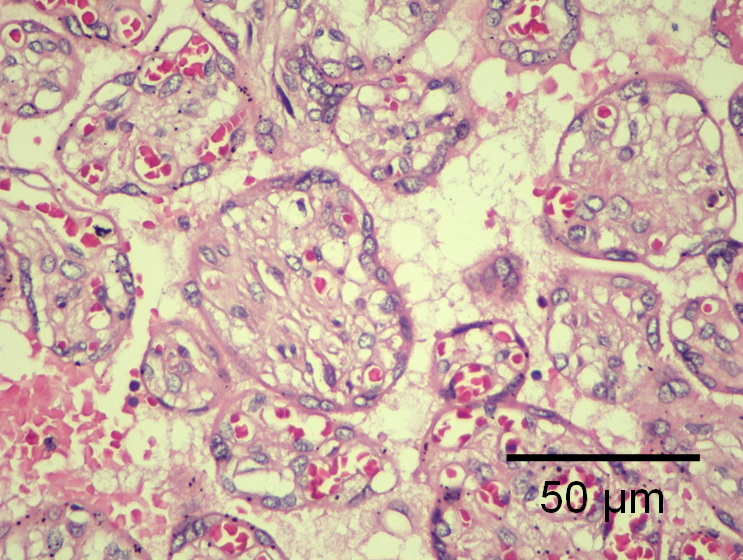


**Figure 2.** Monotrophoblastic placental villi, H&E stain, 400×.


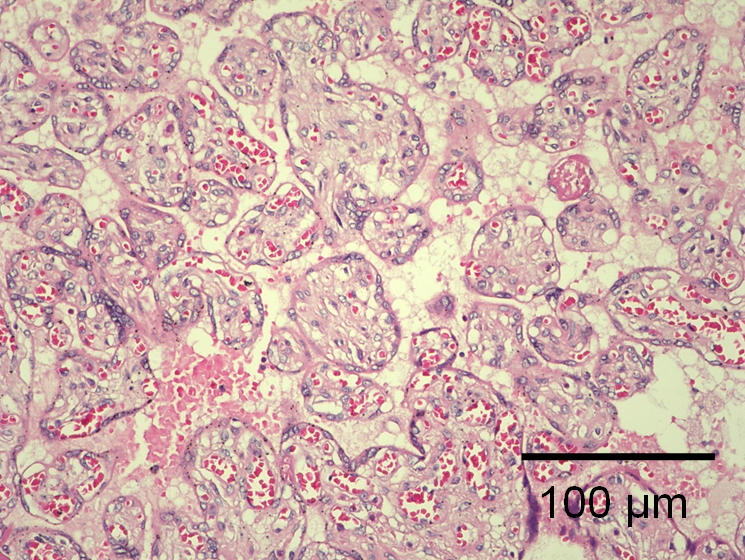


**Figure 3.** Terminal placental villi, H&E stain, 200×.


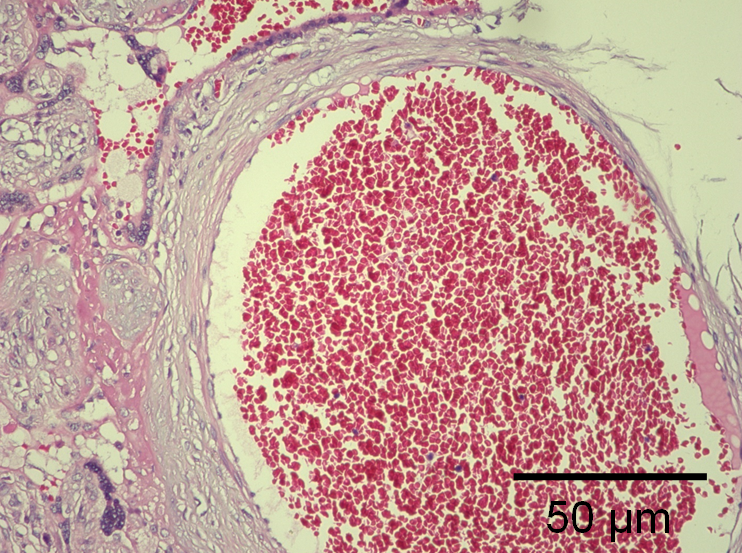


**Figure 4.** Vessel with endothelial discontinuity; partial dissociating edema of the media, H&E stain, 400×.


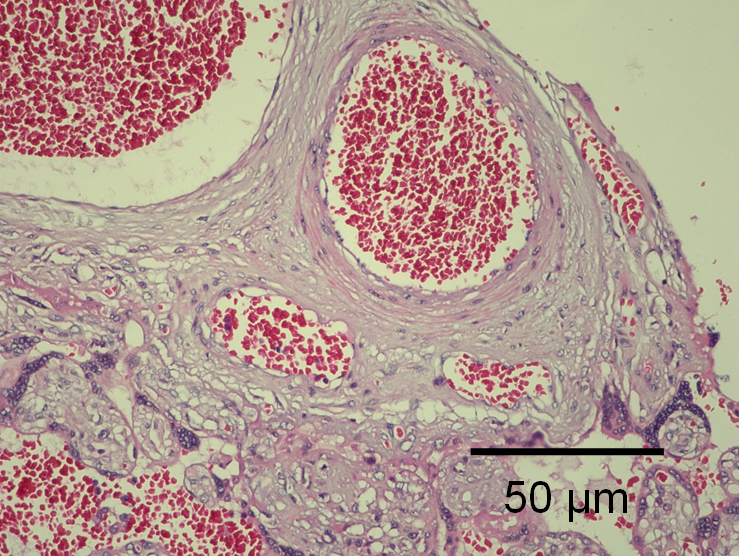


**Figure 5**. Microhemorrhagic foci; ectatic, thrombosed vessels, H&E stain, 400×.


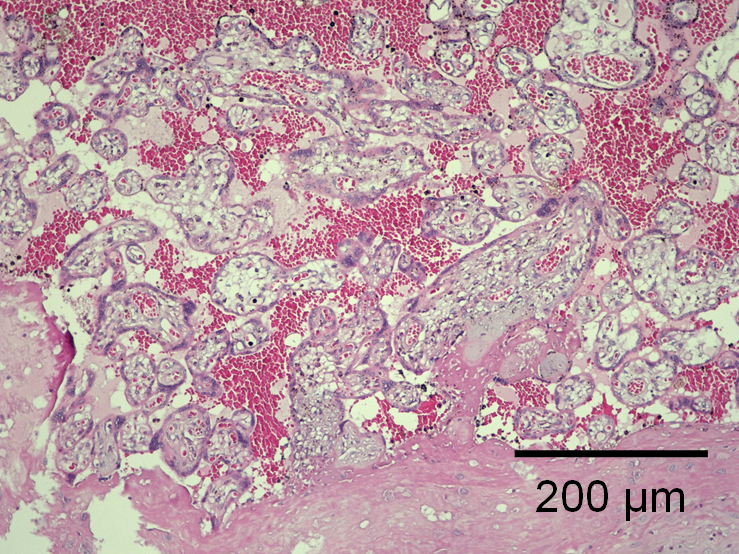


**Figure 6.** Avascular placental villi, H&E stain, 100×.


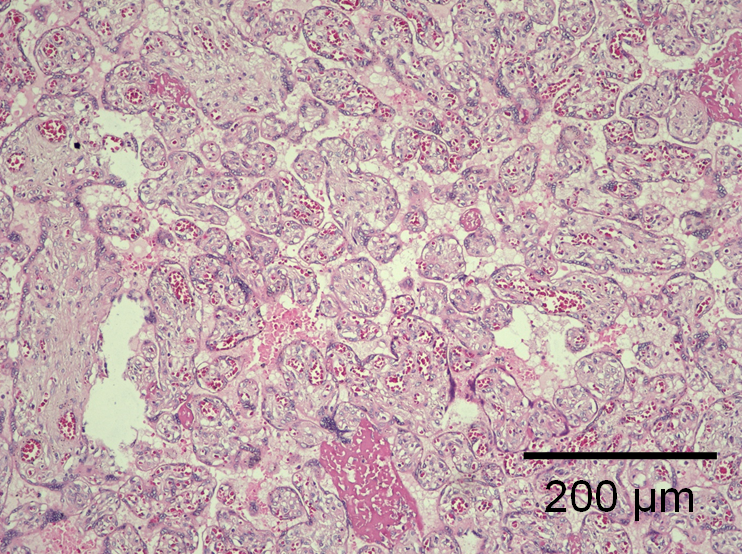


**Figure 7.** Fibrinoid deposits and microhemorrhagic foci, H&E stain, 100×.


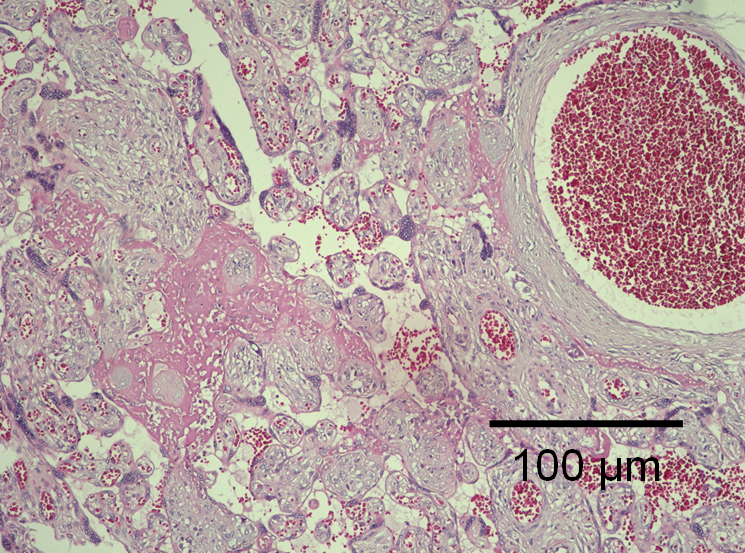


**Figure 8.** Perivillous and intervillous fibrinoid deposits; vascular thrombosis, H&E stain, 200×.


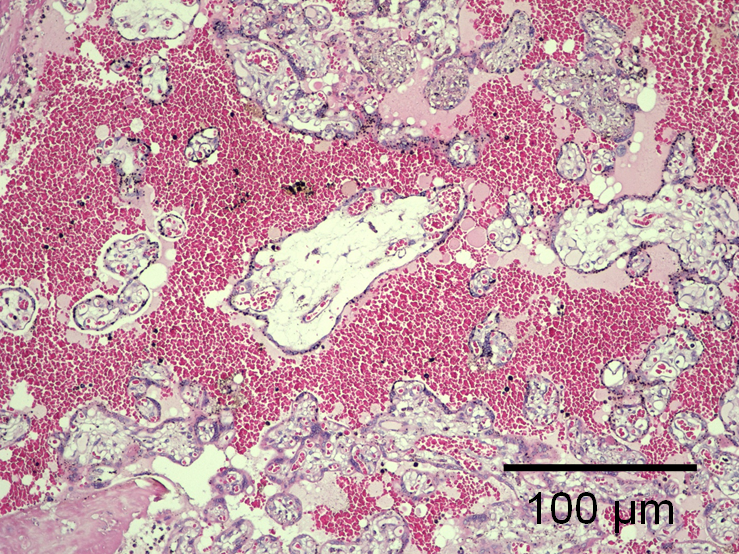


**Figure 9.** Necrobiotic and necrotic-hemorrhagic lesions, H&E stain, 200×.


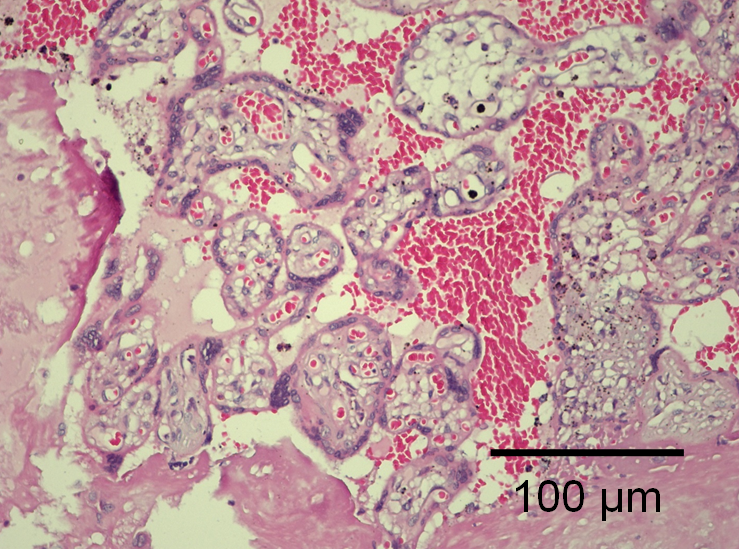


**Figure 10.** Fibrinoid necrosis, isolated calcific deposits, microhemorrhagic foci, H&E stain, 200×.


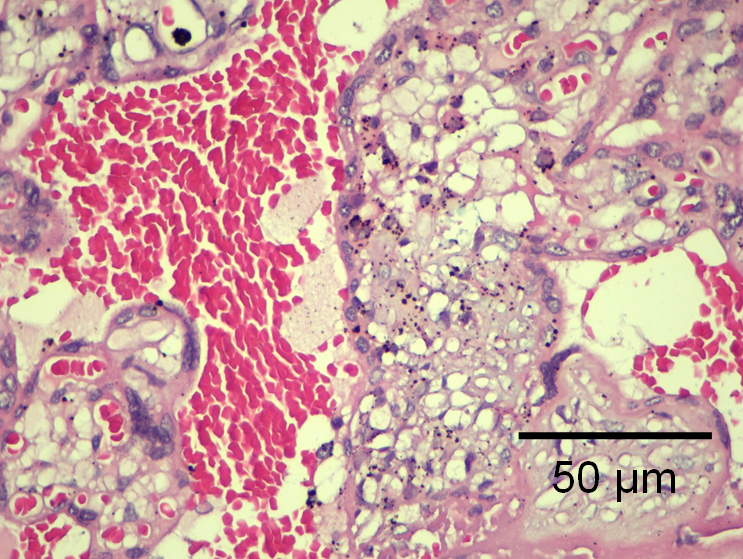


**Figure 11.** Presence of macrophages and hemosiderin deposits, H&E stain, 400×.

Histopathological examination of placental samples revealed a constellation of features consistent with placental immaturity and vascular maladaptation, commonly observed in preterm deliveries. The predominance of immature intermediate villi, together with a well-formed cytotrophoblast and reduced syncytiotrophoblast, indicates delayed villous maturation, a hallmark of premature placental development. Such structural immaturity may impair gas and nutrient exchange, contributing to fetal vulnerability and postnatal respiratory instability, including apnea of prematurity (32). Marked variability in chorionic villi size, reduced cellularity within the villous axis, and stromal laxity further support incomplete placental maturation. These alterations have been associated with reduced functional reserve and diminished adaptive capacity to intrauterine stressors (33). The presence of endothelial discontinuities and pseudoangiomatous features suggests aberrant placental angiogenesis, which may compromise fetoplacental perfusion and oxygen delivery.

Importantly, peri- and intervillous fibrinoid deposits with vascular thrombosis, along with necrotic, hemorrhagic, calcific, and microhemorrhagic lesions, point toward chronic placental hypoperfusion and vascular injury. Such lesions are frequently described in placentas from preterm births and have been linked to adverse neonatal outcomes through impaired placental blood flow and intermittent fetal hypoxia. The identification of hemosiderin-laden macrophages further suggests prior hemorrhagic events and ongoing tissue remodeling, reinforcing the chronicity of placental injury.

Collectively, these findings indicate that structural immaturity and vascular pathology coexist in the examined placentas, providing a plausible pathological substrate for fetal hypoxia and postnatal respiratory dysregulation. While these changes are not specific to apnea of prematurity, they likely contribute to the overall physiological vulnerability observed in affected preterm infants.
