# Supplementary material for: Clinical and biological impact of caffeine therapy in premature infants with apnea of prematurity: a prospective observational study
Source: Front Med (Lausanne). 2026 May 13;13:1808401. doi: 10.3389/fmed.2026.1808401 (PMC13212477; doi:10.3389/fmed.2026.1808401)
Supplement: Supplementary file 2 [file Supplementary_file_2.docx]

**Annex 10.13**

**INFORMED CONSENT OF THE PATIENT**

**on the healthcare provided to the newborn**

This document was issued, concluded and signed under the legal regulations provided for in the POM 16 medical operational procedure.

This document is an annex to the observation sheet and/or the patient's file and is to be attached to it:

The undersigned, (name and surname) ____________________________________________________, residing in (address in C.I.) __________________________________________________________, CNP |__|__|__|__|__|__|__|__|__|__|__|__|__|, C.I./passport series ______, no. __________________, as □ mother / □ father / □ legal representative of the newborn _____________________________, born at ___________ o'clock on the day (day) ________ (month) ___________________ (year) _____________, male □ / female □, admitted to the Neonatology Clinic of the Filantropia Municipal Clinical Hospital in Craiova, declare the following:

- I agree with the diagnostic and treatment procedures applied to my child.
- The nature, purpose, benefits and risks of the procedures applied to my newborn for diagnostic and therapeutic purposes, as well as of the other possible therapeutic options, have been explained to me in my understanding by the pediatric neonatologist, whom I designate to perform, together with the team of assistants in the department, all the necessary procedures.
- I was presented with the associated risks as well as the unpredictable risks (including the risk of death, the risk of sequelae or psycho-motor/neuro-sensory disability and the risk of infection), the consequences, in the short or long term, of the treatment and care provided to the newborn, starting from birth.
- The risks of not performing the treatment were also explained to me; I understood these risks and I accept them.

I certify that I have read and fully understood the above, as well as the information on the back, and therefore sign.

I have been informed that the health care process is doubled by the **educational process** and I consent to participate in the educational process within the limits imposed by decency and common sense, but this must not affect the quality of medical care. I was informed that I can refuse this in principle or at times of my choosing, without being obliged to give any other explanations and without affecting my rights as a patient.

In case of major emergency situations during hospitalization, the medical team is authorized to perform any diagnostic tests or therapeutic maneuvers, medically justified and under the conditions of a correct medical practice.

I agree that the attending physician and any other member of his team **will take and use photographs, video sequences, x-rays and any other clinical data of my case for didactic and scientific documentary purposes**, respecting the confidentiality regarding my identity.

I agree that **my / the patient's identification and contact data should be included in the** hospital's database for the compilation of the observation sheet. My data can be deleted from the database upon express request.

I have acknowledged and agree to respect **the Rules and customs of the hospital**.

Date (day, month, year) ______________________ Signature__________________________

Do you agree with the BCG vaccination? □ YES ____________ / □ NOT ________

Do you agree with the AHB vaccination? □ YES __________ / □ NOT _________

You agree to metabolic screening: PKU, HT, Cystic Fibrosis □ YES __________ / □ NOT _________

**Neonatal pathology and procedures:**

| **NEONATAL PATHOLOGY** | | **PROCEDURES** |
| --- | --- | --- |
| - şoc neonatal - asphyxia, neonatal hypoxia - ischemic hipoxic encephalopathies - Seizures - cerebral hemorrhage (intraventricular, cerebellar, etc.) - leucomalacie periventriculară - retinopathies de prematuritate (ROP) - idiopathic respiratory distress syndrome - respiratory failure - pneumonia - amniotic fluid aspiration syndrome - neonatal transient tachypnea - pulmonary haemorrhage - persistent pulmonary hypertension (PPHT) - heart rhythm disorders - Hypotension - Failure - Freediving Crize - pneumotorax - pulmonary interstitial emphysis - bronhodisplazie pulmonară (BDP) | - Polycythemia - anemia due to incompatibility - anaemia of other maternal-foetal causes, blood group and/or Rh - Hemorrhagic syndrome - Gastrointestinal bleeding - purple - disseminated intravascular coagulation (CID) - jaundice - metabolic disorders (hypo-/hyperglycaemia, hypocalcaemia, hypo-/hypernatremia, hypokalaemia, acidosis) - renal impairment - infection with intrauterine onset (congenital) or postpartum (maternal-fetal and/or nosocomial) infection) - Ulceronecrotic enterocolitis (EUN) - conjunctivitis - malformaţii congenitale - intrauterine growth restriction - prematuritate | **INVASIVE**   - arterial puncture (radial artery) - Percutaneous venous catheterization - umbilical catheterization - CPAP – non-invasive ventilation - IOT – mechanical ventilation - exanghinotransfuzie - Orogastric probe fitting - capillary puncture - lumbar puncture - Paracentesis - Venous puncture / cannula fitting - Transfusion - administration of antibiotics - Electrolyte administration - Immunoglobulin administration - total parenteral feeding - urinary catheter fitting - drenaj pleural   **NON-INVASIVE**   - phototherapy - Radiographs |
|  |  |  |
